# Phenylacetaldehyde synthase 2 does not contribute to the constitutive formation of 2-phenylethyl-β-D-glucopyranoside in poplar

**DOI:** 10.1080/15592324.2019.1668233

**Published:** 2019-09-18

**Authors:** Jan Günther, Axel Schmidt, Jonathan Gershenzon, Tobias G. Köllner

**Affiliations:** Department for Biochemistry, Max Planck Institute for Chemical Ecology, Jena, Germany

**Keywords:** *Populus trichocarpa*, aldehyde synthase, 2-phenylethyl-β-D-glucopyranoside, 2-phenylethanol, herbivory

## Abstract

In response to herbivory, poplar produces among other compounds the volatile alcohol 2-phenylethanol and its corresponding glucoside 2-phenylethyl-β-D-glucopyranoside. While the free alcohol is released only upon herbivory, the glucoside accumulates also in undamaged leaves, but increases after herbivore feeding. Recently we showed that 2-phenylethanol and its glucoside are biosynthesized via separate pathways in *Populus trichocarpa*. The phenylacetaldehyde synthase PtAAS1 plays a central role in the *de novo* formation of herbivory-induced volatile 2-phenylethanol, while the phenylalanine decarboxylase PtAADC1 initiates a pathway responsible for the herbivory-induced production of 2-phenylethyl-β-D-glucopyranoside. Besides PtAAS1, *P. trichocarpa* possesses another aromatic aldehyde synthase PtAAS2 with *in vitro* enzymatic activity comparable to that of PtAAS1. However, in contrast to *PtAAS1*, which is exclusively expressed in herbivory-damaged leaves, *PtAAS2* was found to be expressed at constant levels in both damaged and undamaged leaves. Thus it has been hypothesized that PtAAS2 provides phenylacetaldehyde as substrate for the constitutive formation of 2-phenylethyl-β-D-glucopyranoside in undamaged *P. trichocarpa* trees. By generating RNAi-mediated *AAS2* knockdown plants, we show here that despite the similar activities of PtAAS1 and PtAAS2 *in vitro*, the latter enzyme does not contribute to the biosynthesis of 2-phenylethyl-β-D-glucopyranoside *in planta*. Based on the recent finding that phenylpyruvic acid accumulates in undamaged poplar leaves, the constitutive formation of the glucoside may now be suggested to proceed via the Ehrlich pathway, which begins with the conversion of phenylalanine into phenylpyruvic acid.

To defend themselves against herbivores, plants produce a wide array of natural products, including nonvolatile compounds that accumulate in plant tissue and volatiles that are released upon herbivore attack. These compounds can act as toxins, feeding deterrents, and attractants for predators and parasitoids of the herbivores, and can also warn other plant parts or even neighboring plants of impending herbivory.^1^ Often, plants rapidly biosynthesize such defense compounds after the plant has been damaged.^2^ Poplar trees, for example, produce and emit phenylalanine-derived volatiles and accumulate phenylalanine-derived nonvolatile metabolites upon herbivory by gypsy moth caterpillars (*Lymantria dispar*).^3–6^ The volatile 2-phenylethanol and its corresponding glucoside 2-phenylethyl-β-D-glucopyranoside are major components of this defense reaction.^4^

Recent research showed that the biosynthesis of volatile and glycosidically bound 2-phenylethanol in poplar proceeds via separate pathways that are either initiated by a cytochrome P450 from the CYP79 family, an aromatic aldehyde synthase (AAS), or an aromatic amino acid decarboxylase (AADC), respectively (Figure 1;^3,4^). The AAS and AADC enzymes belong to the class of pyridoxal phosphate group II dependent enzymes, which are known to catalyze diverse enzymatic reactions *in vivo*.^7,8^ The phenylacetaldehyde synthase PtAAS1 and the phenylalanine decarboxylase PtAADC1 have been demonstrated to be involved in 2-phenylethanol formation in western balsam poplar (*Populus trichocarpa*).^4^ *PtAAS1* and *PtAADC1* were found to be expressed in herbivore-damaged *P. trichocarpa* leaves and biochemical characterization of recombinant proteins and RNAi-mediated knockdown experiments revealed that PtAAS1 is involved in the formation of volatile 2-phenylethanol while PtAADC1 provides the substrate for the herbivory-induced production of 2-phenylethyl-β-D-glucopyranoside.^4^

**Figure 1.**
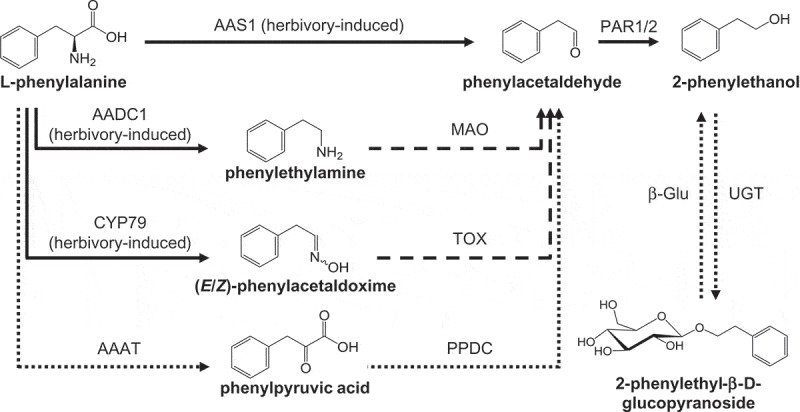
The biosynthesis of 2-phenylethanol and 2-phenylethyl-β-D-glucopyranoside in poplar. AADC, aromatic amino acid decarboxylase; AAS, aromatic aldehyde synthase; MAO, monoamine oxidase; PAR, phenylacetaldehyde reductase; CYP79, cytochrome P450 family 79 enzyme; AAAT, aromatic amino acid transaminase; TOX, transoximase; PPDC, phenylpyruvic acid decarboxylase; UGT, UDP-glucosyl transferase; β-Glu, β-glucosidase. Dashed lines indicate enzymes/reactions not yet characterized *in planta*. Solid lines indicate characterized poplar enzymes, and dotted lines indicate enzymes characterized in other plants.

Besides PtAAS1, another aromatic aldehyde synthase, PtAAS2, was recently identified in *P. trichocarpa*. As PtAAS1, PtAAS2 catalyzes the conversion of L-phenylalanine to phenylacetaldehyde with reasonable kinetic parameters *in vitro*, suggesting that it also acts as a phenylacetaldehyde synthase *in planta*.^4^ However, in contrast to *PtAAS1*, which is strongly expressed in herbivore-damaged leaves, *PtAAS2* showed high expression in undamaged leaves. Thus, it was hypothesized that PtAAS2 produces phenylacetaldehyde as substrate for the constitutive formation of 2-phenylethyl-β-D-glucopyranoside in undamaged *P. trichocarpa* leaves.^4^

To test this hypothesis, we studied the biological function of AAS2 via *Agrobacterium tumefaciens-*mediated RNAi of *PcanAAS2* in gray poplar (*P*. x *canescens*) as described previously.^4^ In brief, sterile stem internodes and leaf material of *P. x canescens* was infiltrated with *A. tumefaciens* carrying an antisense sequence complementary to *PtAAS2*. Transgenic saplings were generated and propagated via callus cultures derived from four independent transgenic events. For comparisons, we included wild type trees and trees transformed with an empty vector into the experiment. Plants were grown until a height of 1 meter and leaves (leaf plastochron index LPI3-10) were harvested for gene expression analysis and liquid chromatography-tandem mass spectrometry (LC-MS/MS) measurements as described in Günther et al.^4^ Real time-quantitative PCR experiments revealed that the expression of *PcanAAS2* was significantly decreased in all *PcanAAS2* RNAi lines in comparison to wild type trees and the empty vector controls (Figure 2a). However, the downregulation of *PcanAAS2* did not influence the accumulation of 2-phenylethyl-β-D-glucopyranoside (Figure 2b). These results suggest, contrary to our original hypothesis, that *AAS2* does not contribute to the constitutive formation of glucosylated 2-phenylethanol in poplar.

**Figure 2.**
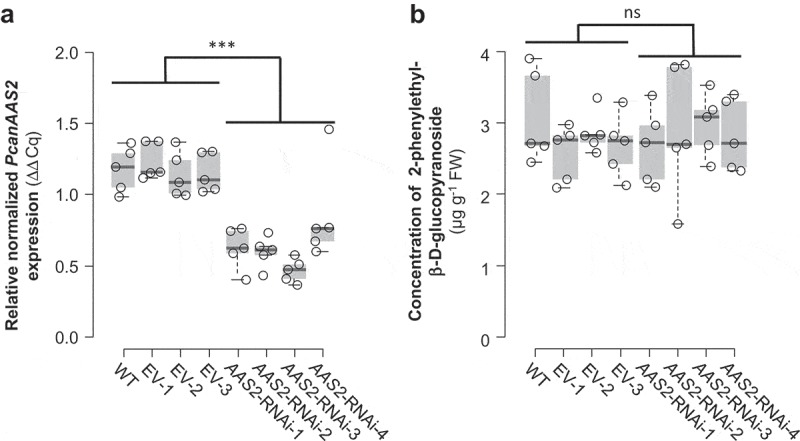
Transcript accumulation of *PcanAAS2* (a) and accumulation of 2-phenylethyl-β-D-glucopyranoside (b) in undamaged leaves of *Populus x canescens* wild type plants (WT), empty vector control plants (EV), and *PcanAAS2* RNAi lines (*AAS2*-RNAi). (a) Gene expression was analyzed using real time-quantitative PCR and the relative normalized expression compared to the reference gene *ubiquitin* is shown. (b) 2-Phenylethyl-β-D-glucopyranoside was extracted with methanol from ground leaf material and analyzed via liquid chromatography-tandem mass spectrometry. Biological replicates (nb) and technical replicates (nt) of EV lines and RNAi lines were used to test for statistical differences. WT, nb = 5; EV, nb = 3, nt = 5; *AAS2*-RNAi, nb = 4, nt = 5 (*AAS2-RNAi-2*, nb = 4, nt = 4). Asterisks indicate statistical significance as assessed by Student’s *t* tests. *PcanAAS2* expression (*P* < 0.001, t = 8.934); 2-phenylethyl-β-D-glucopyranoside accumulation (*P* = 0.792, t = −0.266). Medians ± quartiles and outliers are shown. Each data point is represented by a circle. ns, not significant.

In general, plant aromatic aldehyde synthases can function in various biosynthetic pathways. Our study revealed that PtAAS2 does not play a role in the formation of 2-phenylethyl-β-D-glucopyranoside in poplar. However, the *in vitro* characterization of PtAAS2 showed that this enzyme is able to convert not only phenylalanine into the corresponding phenylacetaldehyde, but also tryptophan into indole-3-acetaldehyde.^4^ Although the kinetic parameters for the conversion of tryptophan have not yet been determined, it is tempting to speculate that PtAAS2 produces mainly indole-3-acetaldehyde *in planta* as a precursor for other tryptophan-derived metabolites. The fact that the expression of *PtAAS2*, in contrast to *PtAAS1*, was not influenced by herbivory further suggests that PtAAS2 is not involved in the defense reaction against herbivores in poplar.

Because PtAAS2 can now be ruled out as candidate for the formation of the basal levels of 2-phenylethyl-β-D-glucopyranoside in undamaged poplar leaves, it is likely that another pathway provides the required phenylacetaldehyde substrate in these leaves. The stable accumulation of phenylpyruvic acid in undamaged and damaged *P. trichocarpa* leaves^4^ indicates that the Ehrlich pathway might play a role in constitutive 2-phenylethyl-β-D-glucopyranoside accumulation. The Ehrlich pathway was originally elucidated in yeast and requires the action of an aromatic amino acid transaminase (AAAT) and a phenylpyruvic acid decarboxylase (PPDC) (Figure 1; for review see Hazelwood et al.^9^). Both enzymes have been identified in melon^10^ and rose^11^ and it would thus be worthwhile to investigate potential AAAT and PPDC candidates in future studies of 2-phenylethyl-β-D-glucopyranoside biosynthesis in poplar.
